# Influence of vitamin D supplementation on immune function of healthy aging people: A pilot randomized controlled trial

**DOI:** 10.3389/fnut.2022.1005786

**Published:** 2022-11-01

**Authors:** Honglin Dong, Viktorija Asmolovaite, Sebastien Farnaud, Derek Renshaw

**Affiliations:** ^1^School of Life Sciences, Coventry University, Coventry, United Kingdom; ^2^Centre for Sport, Exercise and Life Sciences, Coventry University, Coventry, United Kingdom

**Keywords:** vitamin D, aging, immune function, proinflammatory cytokines, creatinine, randomized controlled trial

## Abstract

**Objectives:**

This study aims to investigate the influence of vitamin D supplementation on immune function of healthy older adults.

**Materials and methods:**

Designed as a randomized controlled trial, 21 participants (55–85 years) completed the study during May–November 2018 in Coventry, England. The participants were randomized into vitamin D or the control group, stratified by age, gender and body mass index. The vitamin D group (*n* = 12) took vitamin D3 tablets of 1,000 IU/day for 12 weeks plus vitamin D education leaflet, while the control group (*n* = 9) were only provided with the leaflet. At baseline, 6 and 12 weeks, plasma 25(OH)D levels and immunological and metabolic parameters including phagocytic activity of granulocytes and monocytes, tumor necrosis factor, interleukin 6, lymphocyte subsets and fasting blood glucose and lipid were measured. Dietary vitamin D intake was analyzed at baseline and week 12. Data were presented as mean ± SD. Two-way repeated measures ANOVA and independent *t*-test were used to analyze the data.

**Results:**

At baseline, 42.9% of the participants were vitamin D deficiency (25(OH)D < 25 nmol/L), only 10% achieved a level of 25(OH)D > 50 nmol/L. Overweight/obese participants (*n* = 9) had significantly lower mean plasma 25(OH)D concentration (22.3 ± 8.7 nmol/L) than normal weight participants (48.1 ± 34.3 nmol/L) (*P* = 0.043). There was a significant increase in plasma 25(OH)D concentration in vitamin D group compared with that in control group (*P* = 0.002) during the intervention period. The plasma 25(OH)D concentration in vitamin D group was increased at 6 weeks (from 38.4 ± 37.0 nmol/L at baseline to 51.0 ± 38.2 nmol/L) with little change observed between 6 and 12 weeks (51.8 ± 36.4 nmol/L). The plasma creatinine concentration in vitamin D group was significantly decreased compared with the control group (*P* = 0.036) (79.8 ± 7.0 μmol/L at baseline vs 75.1 ± 5.4 μmol/L at week 12). No significant effect of vitamin D supplementation was determined on immunological parameters.

**Conclusion:**

Vitamin D deficiency is common among the aging population in the UK even during the summertime. Vitamin D supplementation at 1,000 IU/day for 12 weeks significantly increased plasma 25(OH)D concentration but showed no effect on metabolic and immunological parameters except decreased plasma creatinine.

## Introduction

Apart from its classic role in promoting calcium absorption in the gut, vitamin D (VitD) also plays important roles in modulation of cell growth, neuromuscular and immune function and reduction of inflammation due to its receptors expressed ubiquitously in nearly all human body cells, including B cells, T cells and dendritic cells (1). *In vitro* experiments show that 1,25(OH)2D (the active form of VitD) enhances the effects of macrophages and monocytes against pathogens by stimulating the differentiation of monocytes into mature phagocytic macrophages (2), induces anti-bacterial peptide cathelicidin and β-defensin 2 production (3), and has an anti-inflammatory effect, shifting of T-cells to preferential Th2 cell development with interleukin 4 (IL-4), Interleukin 5 (IL-5), and interleukin 10 (IL-10) production associated with antibody mediated humoral immunity over Th1 development with interferon-gamma (IFN-γ) production (4). Observational studies also show there was an association of VitD deficiency and increased risk of autoimmune diseases such as multiple sclerosis, rheumatoid arthritis, type 1 diabetes mellitus and infection rate (1), and an inverse association of VitD status (defined by blood 25(OH)D concentration) with inflammatory status in older people (5, 6).

The decline in immune function is one of the most recognized consequences of aging, which contributed to the increased vulnerability to infection including influenza (7, 8). Specific changes to immune function include a decline in innate immunity including phagocytic activity and individual natural killer (NK) cell activity, decreases in the numbers of T cells and its capacity to proliferate and secrete cytokines, and specific antibody responses (9), a reversal of CD4/CD8 T-cell ratio, and a lingering level of low-grade inflammation termed “inflamm-aging” (10).

VitD deficiency is common in older adults due to low dietary VitD intake (11), reduced outdoor activities, and declined production of VitD in the skin and reduced ability to make the active form of VitD as part of the aging process (12). The elderly only produce about 25% of the VitD as compared to young adults at 20 years old after exposure to the same amount of sunlight (13). It has been reported that the overall prevalence of VitD deficiency in England at high latitude (52.3555° N, 1.1743° W) was 26.4% defined as 25(OH)D < 30 nmol/L by Institute of Medicine (IOM) and 58.7% defined as 25(OH)D < 50 nmol/L by the Endocrine Society (ES) (14).

The evidence of the effect of oral VitD supplementation on immune function in older adults is ambiguous. Therefore, this pilot randomized controlled trial was devised aiming to investigate the influence of VitD supplementation at 1,000 IU/day for 12 weeks on the immune function of the free living healthy older adults.

## Materials and methods

The study was approved by Coventry University Ethics Committee (Reference no. P66509) and was performed during May–November 2018 in Coventry, England, in accordance with the ethical standards laid down in the 1964 Declaration of Helsinki. All participants gave their written consent before taking part in the study.

### Participants

The inclusion criteria of the study were 55–85 years old, both male and female and in general good health. The exclusion criteria were ongoing inflammatory or infectious diseases, autoimmune diseases, malignancy, cirrhosis, digestive disease, use of anti-inflammatory or immunomodulating medications (including oral prednisone and other steroids) currently or within 4 weeks prior to participation of the study, consumption of VitD supplement more than 400 IU/day, regular smoker (≥1 cigarette/day), alcoholism and drug misuse, vegetarian or vegan (vitamin D3 supplement tablets were from animal sources). The participants were recruited through local library, AGE UK, and newspaper advertisement with gatekeeper permissions. A health and lifestyle questionnaire (Supplementary Table 1) was used to screen the eligibility of the participants.

### Study design

The study was designed as a paralleled randomized controlled trial. The participants were randomized by an Excel-based covariate adaptive randomization programme (15) to enter two intervention arms: VitD supplementation and control group, stratified by age (3 levels, 55–65 years, 66–75 years, and 76–85 years), gender (2 levels, female and male), and body weight categories defined by body mass index (BMI) (3 levels, normal weight: 18–24.9 kg/m^2^, overweight: 25–29.9 kg/m^2^, and obese: ≥ 30 kg/m^2^) (16). The VitD group took VitD3 supplement tablets (Holland & Barrett International Ltd., Nuneaton, UK) at 1,000 IU/day (one tablet per day) for 12 weeks plus provision of education leaflet of VitD, while the control group were only provided with education leaflet of VitD. We chose VitD supplementation of 1,000 IU/day (25 μg/day) for 12 weeks mainly based on the literature that anti-fall benefits of VitD supplementation were observed from a dose of 700 IU/day onwards and recommended a higher dose to lead to positive effects of VitD supplement on immune function (17). In addition, evidence shows VitD supplementation with 800 IU/day raised the mean serum 25(OH)D concentration significantly at 3 months and was kept stable up to 2 years of supplementation (18). At baseline (visit 1 or V1), in the middle of the intervention (visit 2 or V2 after 6 weeks) and at the end of the intervention period (visit 3, V3 after 12 weeks), fasting blood samples were collected via phlebotomy into sodium heparin vacutainer tubes stored on ice for immunological and biochemical analysis. The body weight and height (the height was measure only at V1), waist circumference, blood pressure and heart rate were also measured at each visit. A 4-day estimated food diary was collected at V1 and V3 only.

### Plasma separation

Blood samples (0.5 ml) were set aside for immune parameter measurement and the remaining blood was centrifuged at 2,000 × *g* for 15 min at 4°C. Aliquots of plasma were stored at -20°C for later analysis.

### Immune parameters

Phagocytic activity of leucocytes: Phagocytic activity of granulocytes and monocytes were determined by a commercial kit (BD Biosciences, Franklin Lakes, NJ, USA) using whole blood. The percentage of granulocytes or monocytes engaged in phagocytosis of *Escherichia coli* was detected by BD Accuri C6 flow cytometer (BD Biosciences, Franklin Lakes, NJ, USA) within 24 h. The laboratory procedure followed the manufacturer’s instructions. Data were analyzed using Accuri C6 Software (BD Biosciences, Franklin Lakes, NJ, USA).

Lymphocyte phenotypes: Whole blood was stained with appropriate combinations of fluorescently labeled mouse anti-human monoclonal antibodies (BD Biosciences, Franklin Lakes, NJ, USA) for discrimination between different lymphocyte subsets, including T lymphocytes (CD3^+^), helper T cells (Th, CD3^+^CD4^+^), cytotoxic T cells (Tc, CD3^+^CD8^+^), NK cells (CD3^–^CD56^+^), CD8^+^ NK cells (CD3^–^CD8^+^CD56^+^) and CD8^–^ NK cells (CD3^–^CD8^–^CD56^+^). The steps of procedure followed the manufacturer’s instruction. The samples were detected by BD Accuri C6 flow cytometry within 24 h. The lymphocytes were gated, and fluorescence data for 10,000 events were collected and analyzed by Accuri C6 Software. Results of phenotypes were expressed as the percentage of the cell group specified.

### Plasma 25(OH)D measurement

VitD status was evaluated by the measurement of plasma 25(OH)D concentrations using Tosoh AIA-900 immunoassay analyzer (Tosoh Bioscience, USA) following the manufacturers instruction. The Tosoh ST AIA-PACK 25(OH)D assay correlates well with gold standard methods (Liquid chromatography–mass spectrometry, LC-MS), measures 25(OH)D2 and 25(OH)D3 in equimolar proportions and aligns to the reference measurement procedure used in the VitD standardization program (VDSP) (19). The quality control was in place to verify that the result obtained was within the range of expected values. Assay range of 25(OH)D was between 10 and 300 nmol/L.

### Plasma interleukin 6 (IL-6) and tumor necrosis factor (TNF) measurement

Plasma interleukin 6 (IL-6) and tumor necrosis factor (TNF) were measured by ELISA kits (BD Biosciences, Franklin Lakes, NJ, USA) following the manufacturer’s instruction. The minimum detectable concentration was 2.2 pg/ml for IL-6 and 2.0 pg/ml for TNF. The average intra and inter coefficient of variation (CV) was 6.7 and 9.4% for IL-6, and 3.7 and 6.1% for TNF. The results were analyzed by using GraphPad Prisma (Version 9.3.1) (GraphPad Software, San Diego, CA, USA).

### Plasma metabolic parameters

Plasma glucose, cholesterol, high density lipoprotein (HDL), calcium, and creatinine were tested by Horiba Pentra 400 Biochemistry Analyzer (Horiba, Japan) following the manufacturer’s instruction. The detection limit was 0.11 mmol/L for glucose, 0.10 mmol/L for cholesterol, 0.03 mmol/L for HDL, 0.07 mmol/L for calcium, and 19.5 μmol/L for creatinine.

### Side effect monitor

Plasma calcium and creatinine were measured at each visit, and any symptoms during the intervention period were recorded upon report from participants.

### Compliance

A total of 100 tablets of VitD3 in one bottle were provided to participants in the VitD group. They were asked to log their consumption of VitD3 tablets on a daily basis and return the bottle and the log at their last visit (V3). The left-over tablets were counted combined with the log to monitor the compliance. Sixteen tablets per bottle were expected to be remaining if the participant was fully compliant.

### Food diary analysis

Participants were asked to record a 4-day (consecutive days, including a weekend day) estimated food diary. Food recording with a minimum of 3 days is regarded as a gold standard method to assess nutrient intake (20). A template food diary with an example and guidance was provided to participants. Completed food diaries were collected via email or hard copy and dietary VitD intake was analyzed using the nutrition analytical software Nutritics (Nutritics Ltd., Dublin, Ireland). Food diaries that were incomplete or with an energy intake ≤ 1,000 kcal/day or ≥ 4,000 kcal/day were excluded. This was to address the issue of implausible energy intake that might indicate inaccuracy in the food record (21). In this study none of the food diaries collected were excluded as they were all within the expected calorie range.

### Statistical analysis

Quantitative variables were presented as mean ± standard deviation (SD). Categorical variables were presented as percentage (%), e.g., % of VitD deficiency. Statistical analysis was performed using SPSS software (Version 26). Differences in quantitative variables between VitD and control groups over the 12 weeks of intervention period were analyzed by using two-way repeated measures ANOVA, illustrated by the main effect of visit*treatment interaction with age as a covariate. Baseline quantitative variables between the two groups were analyzed by independent *t*-test. Pearson correlation was used to find the association between two quantitative variables. The statistically significant level was set up at *P* ≤ 0.05 with two-tail.

## Results

### Basic information of participants

**FIGURE 1:**
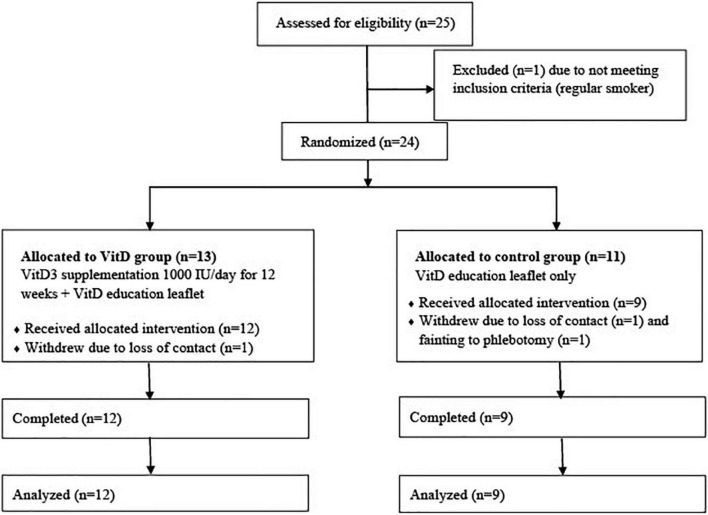
CONSORT flow chart of participants through the trial. VitD, vitamin D.

Twenty-five participants were approached and one was ineligible for the study due to smoking. Twenty-four eligible participants were recruited and randomized to VitD (*n* = 13) and control (*n* = 11) group, but one from the VitD group, one from the control group lost contact before they started the study, and one participant from the control group withdrew after the first visit due to fainting during phlebotomy. A total of 21 participants (55–85 years) took part and all completed the study (Figure 1).

Table 1 shows the basic information of the participants. There were 9 participants in control and 12 in VitD group. The average age of the participants was (65.8 ± 7.8) years old (67.4 ± 8.1 years in control group vs. 64.5 ± 7.6 years in VitD group). All were white Caucasian (*n* = 20) apart one from South Asia (Indian ethnicity). Among all participants, 76% (*n* = 16) were females, 24% (*n* = 5) were males; 57% (*n* = 12) had normal weight, and 43% (*n* = 9) were overweight or obese. There were more overweight/obese participants in VitD group than in control group (58 vs 22%).

**TABLE 1:** Characteristics of the participants.

		Total	VitD	Control
All participants (*n*)		21	12	9
Age (years, mean ± SD)		65.8 ± 7.8	64.5 ± 7.6	67.4 ± 8.1
Gender	Female (*n*)	16	9	7
	Male (*n*)	5	3	2
BMI	Normal weight (*n*)	12	5	7
	Overweight/obese (*n*)	9	7	2
Ethnicity	White Caucasian (*n*)	20	12	8
	South Asian (*n*)	1	0	1

BMI, body mass index; Normal weight, BMI 18.5–24.9 kg/m^2^; Overweight/obese, BMI ≥ 25 kg/m^2^. VitD, vitamin D.

### Baseline data results

Table 2 shows the measures of metabolic and immunological parameters of the participants at baseline. Table 3 demonstrates the association of plasma 25(OH)D with the metabolic and immunological parameters at baseline. There was a significant inverse association of plasma 25(OH)D concentrations with BMI (*r* = -0.517, *P* = 0.016) and a significant positive association of plasma 25(OH)D with plasma calcium concentration (*r* = 0.454, *P* = 0.039). There was no significant association of plasma 25(OH)D with other parameters.

**TABLE 2:** Baseline measures of the participants.

	Total	
	
	Mean	SD
BMI (kg/m^2^)	25.2	4.0
Waist circumference (cm)	88.5	11.7
Systolic blood pressure (mmHg)	131.4	23.0
Diastolic blood pressure (mmHg)	77.1	9.4
Heart rate (beat/minute)	61.7	8.8
Fasting glucose (mmol/L)	5.0	0.2
Fasting cholesterol (mmol/L)	5.7	1.5
Fasting HDL (mmol/L)	1.6	0.3
Ratio of cholesterol/HDL	3.7	1.0
Plasma calcium (mmol/L)	2.3	0.1
Plasma creatinine (μmol/L)	75.5	10.5
Plasma 25(OH)D (nmol/L)	37.0	29.3
Plasma TNF (pg/ml)	4.0	4.2
Plasma IL-6 (pg/ml)	9.0	3.0
Phagocytosis of granulocytes (%)	96.6	3.4
Phagocytosis of monocytes (%)	91.6	2.5
CD3^+^CD4^+^ (% lym)	65.8	9.5
CD3^+^CD8^+^ (% lym)	27.6	8.1
CD4^+^/CD8^+^	2.7	1.1
CD3^–^CD56^+^ cells (% lym)	7.4	3.6
CD8^+^CD56^+^ (% CD3^–^ lym)	9.7	5.6
CD8^–^CD56^+^ (% CD3^–^ lym)	12.6	7.0

BMI, body mass index; HDL, high density lipoprotein; TNF, tumor necrosis factor; IL-6, interleukin 6; Th, helper T cells; Tc, cytotoxic T cells; lym, lymphocytes; VitD, vitamin D.

**TABLE 3:** Baseline association of plasma 25(OH)D concentrations with different parameters.

Plasma 25(OH)D (nmol/L)	*r*	df	*P*-value*
BMI (kg/m^2^)	–0.517	19	0.016*
Waist circumference (cm)	–0.364	19	0.105
Systolic blood pressure (mmHg)	–0.078	19	0.738
Diastolic blood pressure (mmHg)	0.076	19	0.742
Heart rate (beat/minute)	0.195	19	0.397
Fasting plasma glucose (mmol/L)	0.038	19	0.872
Fasting plasma cholesterol (mmol/L)	0.24	19	0.294
Fasting HDL (mmol/L)	0.123	19	0.594
Ratio of cholesterol/HDL	0.145	19	0.531
Plasma calcium (mmol/L)	0.454	19	0.039*
Plasma creatinine (μmol/L)	0.138	19	0.551
Plasma TNF (pg/ml)	–0.03	18	0.896
Plasma IL-6 (pg/ml)	–0.129	18	0.588
Phagocytosis of granulocytes (%)	0.184	19	0.438
Phagocytosis of monocytes (%)	0.22	19	0.337
Th cell (CD4^+^% lym)	0.213	19	0.255
Tc cell (CD8^+^% lym)	–0.263	19	0.249
CD4/CD8 ratio	0.226	19	0.324
CD3^–^CD56^+^ cells (% lym)	–0.101	19	0.664
CD8^+^CD56^+^ (% CD3^–^ lym)	0.116	19	0.618
CD8^–^CD56^+^ (% CD3^–^lym)	0.112	19	0.629
Dietary VitD intake (μg/day)	0.328	19	0.146

*Pearson correlation.

BMI, body mass index; HDL, high density lipoprotein; TNF, tumor necrosis factor; IL-6, interleukin 6; Th, helper T cells; Tc, cytotoxic T cells; lym, lymphocytes; VitD, vitamin D.

Table 4 shows the VitD status of the participants at baseline. VitD status was categorized based on the plasma 25(OH)D concentration as deficient (<25 nmol/L), insufficient (25–50 nmol/L, excluding 50 nmol/L), sufficient (≥50 nmol/L) recommended by SACN (22), and optimal or desirable (75–125 nmol/L) according to guidelines by the Institute of Medicine (IOM) (23). The average plasma 25(OH)D of all participants was 37.0 ± 29.3 nmol/L. Around 33% of the participants were VitD deficient, 57% were VitD insufficient, and only 10% were VitD sufficient. There was no significant difference in VitD status between genders, however, the plasma 25(OH)D concentration was significant lower in overweight/obese participants (22.3 ± 8.7 nmol/L) than in normal weight participants (48.1 ± 34.3 nmol/L) (*P* = 0.043). Around 55.6% of overweight/obese participants were VitD deficient vs. 16.7% of the normal weight.

**TABLE 4:** Vitamin D status at baseline.

	Plasma 25(OH)D (nmol/L)	Deficiency *n* (%)	Insufficiency *n* (%)	Sufficiency *n* (%)
Total (*n* = 21)	37.0 ± 29.3	7 (33.3%)	12 (57.1%)	2 (9.5%)
Female (*n* = 16)	39.0 ± 33.2	5 (31.3%)	9 (56.3%)	2 (12.5%)
Male (*n* = 5)	30.6 ± 10.5	1 (20.0%)	4 (80%)	0
Normal weight (*n* = 12)	48.1 ± 34.3*	2 (16.7%)	8 (66.7%)	2 (16.7%)
Overweight/obese (*n* = 9)	22.3 ± 8.7	5 (55.6%)	4 (44.4%)	0

*P = 0.043 independent t-test compared with overweight/obese group.

Data were presented as mean ± SD.

Vitamin D deficiency, plasma 25(OH)D < 25 nmol/L; insufficiency, plasma 25(OH)D 25-50 nmol/L (excluding 50 nmol/L); sufficiency, plasma 25(OH)D ≥ 50 nmol/L.

No significant difference was observed in dietary VitD intake before and after the intervention among all participants or between groups (Table 5). Two food diaries were not collected after the intervention, one from control and one from VitD group. Only two participants achieved taking more than 10 μg/day before the intervention (13.5 and 12.2 μg/day, respectively), and two participants after the intervention (11.0 and 11.1 μg/day, respectively). The main dietary source of VitD were from Horlicks Light Malt instant powder, salmon, multivitamin tablets (VitD less than 5 μg/day), and cod liver oil.

**TABLE 5:** Dietary vitamin D intake before and after the intervention.

	Before intervention (μ g/day)	After intervention (μ g/day)
All participants	3.7 ± 3.7	3.0 ± 3.7
Control	3.6 ± 3.9	2.5 ± 2.7
VitD	3.8 ± 3.5	3.4 ± 4.2

Data were presented as mean ± SD. VitD, vitamin D.

### Intervention data results

**FIGURE 2:**
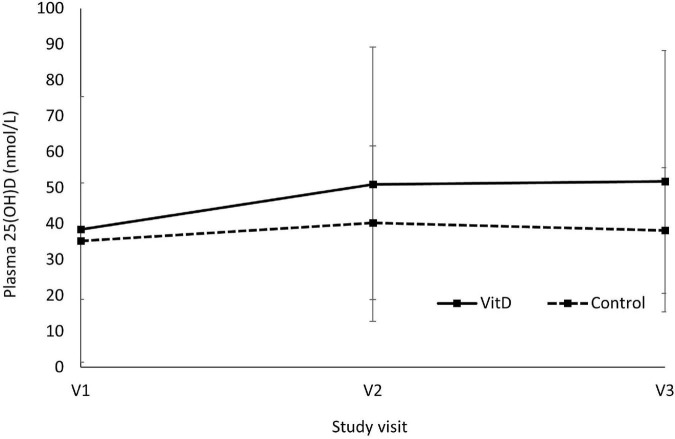
Changes of plasma 25(OH)D concentrations between vitamin D and control group. V1 = visit 1, baseline; V2 = visit 2, after 6 weeks; V3 = visit 3, after 12 weeks. Main effect of visit, F (2,34) = 17.670, *P* < 0.001, ηp^2^ = 0.510; Visit*Treatment interaction, F (2,34) = 9.214, *P* = 0.001, ηp^2^ = 0.352; V1 vs. V2, *P* < 0.001; V1 vs. V3, *P* < 0.001; V2 vs. V3, *P* = 1.00, using two-way repeated measures ANOVA.

Figure 2 shows the changes of plasma 25(OH)D concentrations between VitD and control group. There was a significant main effect of visit (*F* (2,34) = 17.670, *P* < 0.001, ηp^2^ = 0.510) and visit*treatment interaction (F (2,34) = 9.214, *P* = 0.001, ηp^2^ = 0.352) on plasma 25(OH)D concentrations during the study period. Plasma 25(OH)D was significantly increased in VitD group from 38.4 ± 37.0 nmol/L at baseline (V1) to 51.0 ± 38.2 nmol/L after 6 weeks (V2), and 51.8 ± 36.4 nmol/L after 12 weeks (V3) in VitD group, while this has remained stable during the intervention period in control group. There was a significant increase in plasma 25(OH)D between baseline and V2 (*P* < 0.001), and between baseline and V3 (P < 0.001). However, there was no changes of plasma 25(OH)D between V2 and V3 (*P* = 1.00). Only one participant in the VitD group achieved the level of 25(OH)D higher than 75 nmol/L after 12 weeks of VitD3 supplementation (162 nmol/L), however, the baseline level of the participant was very high (149 nmol/L) too. This might be responsible for the high SD in the VitD group.

No significant effect was observed on the metabolic parameters apart from plasma creatinine concentration, which shows a significant decrease in VitD group vs. control group (*F* (2, 36) = 4.283, ηp^2^ = 0.192, *P* = 0.021) (Table 6). However, there was a significant higher plasma creatinine concentration in VitD group than in control group (79.53 ± 7.0 vs 69.9 ± 12.1 μmol/L, *P* = 0.029) at baseline (V1) (Table 6). All participants had normal plasma creatinine concentrations (less than 100 μmol/L (24), however, 5 out of 6 participants who had baseline plasma creatinine concentration more than 80 μmol/L (range of 82.2–89.8 μmol/L) happened to be in the VitD groups (data not shown). There were no significant differences in other parameters between the two groups at baseline.

**TABLE 6:** Metabolic parameters at different visits of the study.

Parameters	Group	V1		V2		V3		*P*-value*
				
		Mean	SD	Mean	SD	Mean	SD	(visit*treatment interaction)
BMI	Control	23.9	3.5	24.0	3.6	24.0	3.7	0.266
	VitD	26.3	4.2	26.1	4.1	26.3	4.4	
WC	Control	86.2	11.5	87.7	14.1	86.6	13.6	0.749
	VitD	90.2	12.1	90.4	13.3	90.4	14.6	
SBP	Control	136.9	25.1	136.8	21.8	136.6	20.8	0.957
	VitD	127.3	21.5	130.3	16.5	127.8	21.3	
DBP	Control	78.2	6.1	78.6	10.9	78.3	7.4	0.920
	VitD	76.3	11.5	77.3	8.9	77.8	9.1	
HR	Control	61.4	7.9	60.9	8.9	64.4	9.8	0.914
	VitD	61.9	9.8	59.8	8.1	64.8	13.6	
Glucose	Control	5.0	0.3	5.1	0.4	5.2	0.4	0.497
	VitD	5.0	0.2	5.0	0.3	5.2	0.3	
Cholesterol	Control	6.0	1.8	6.1	1.5	6.0	1.6	0.491
	VitD	5.6	1.2	5.9	1.4	5.8	1.2	
HDL	Control	1.6	0.3	1.5	0.3	1.6	0.2	0.199
	VitD	1.5	0.4	1.6	0.4	1.6	0.4	
CHDLratio	Control	3.7	1.2	4.0	1.2	3.8	1.2	0.465
	VitD	3.7	0.8	3.8	0.9	3.8	0.8	
Calcium	Control	2.29	0.1	2.31	0.1	2.33	0.1	0.831
	VitD	2.24	0.1	2.26	0.1	2.26	0.1	
Creatinine	Control	69.9	12.1	70.4	12.5	70.5	11.5	0.021**
	VitD	79.8*	7.0	77.7	7.7	75.1	5.4	

*P = 0.029, independent t-test compared with control group; **two-way repeated measures ANOVA; BMI, body mass index; WC, waist circumference; SBP, systolic blood pressure; DBP, diastolic blood pressure; HR, heart rate; HDL, high density lipoprotein; CHDLratio, cholesterol and HDL ratio; VitD, vitamin D.

No significant effect of visit*treatment interaction was observed on the immunological parameters over the intervention period (Table 7).

**TABLE 7:** Immunological parameters at different visits of the study.

Parameters	Group	V1		V2		V3		*P*-value*
				
		Mean	SD	Mean	SD	Mean	SD	(visit*treatment interaction)
TNF (pg/ml)	Control	2.5	3.3	2.3	2.5	4.7	2.6	0.450
	VitD	5.2	4.7	4.9	6.2	4.9	3.6	
IL-6 (pg/ml)	Control	8.4	0.9	8.5	0.9	9.5	3.7	0.296
	VitD	9.5	4.0	11.3	6.0	8.3	0.6	
Phagocytosis of Granulocytes (%)	Control	96.9	2.2	95.8	3.6	96.3	4.0	0.601
	VitD	96.9	4.2	97.5	1.3	98.1	0.8	
Phagocytosis of Monocytes (%)	Control	91.7	2.0	86.3	5.9	88.9	4.2	0.807
	VitD	91.5	2.8	87.7	4.6	90.2	3.3	
CD3^+^CD4^+^ (% lym)	Control	65.0	12.2	66.5	9.2	66.5	9.0	0.692
	VitD	66.4	7.3	65.6	9.5	65.7	7.9	
CD3^+^CD8^+^ (% lym)	Control	28.1	9.8	28.8	9.3	29.1	9.1	0.990
	VitD	27.2	7.1	28.2	7.0	28.3	7.2	
CD4/CD8	Control	2.6	1.2	2.6	1.0	2.6	1.0	0.168
	VitD	2.7	1.0	2.5	1.0	2.5	0.9	
CD56 (% lym)	Control	8.6	4.5	9.6	3.2	10.9	2.5	0.212
	VitD	6.6	2.6	8.2	4.3	7.2	3.4	
CD8^+^CD56^+^ (% CD3^–^ lym)	Control	10.2	6.4	12.7	5.7	12.5	5.1	0.524
	VitD	9.4	5.2	12.3	6.3	9.9	5.0	
CD8^–^CD56^+^ (% CD3^–^ lym)	Control	14.2	8.4	16.1	6.0	19.6	8.1	0.212
	VitD	11.5	5.9	13.9	6.9	13.2	8.0	

*Two-way repeated measures ANOVA.

TNF, tumor necrosis factor; IL, interleukin; lym, lymphocytes; VitD, vitamin D.

### Side effects and safety issues

No cases of high plasma calcium (defined as a concentration ≥2.60 mmol/L) were determined during the study (range of calcium was 2.03–2.48 mmol/L). One participant in VitD group reported mild stomach upset in three occasions during the study, all happened during running. The plasma calcium concentrations for the three visits of the participant were normal (2.32, 2.26, and 2.40 mmol/L, respectively). The participant did not cease taking VitD supplement tablets until completing the study.

### Compliance

The participants were required to consume 84 VitD supplement tables in total for the study. The mean reported VitD supplement intake was 83 tablets based on the log, while the actual intake was 81 tablets according to the tablets left (Supplementary Table 2). Twenty-five percentage of the participants consumed tablets between 70 and 79, 75% of them consumed more than 80 tablets.

## Discussion

This pilot study shows that VitD deficiency is common among this older population (33%) even during the summertime (the study was conducted during May–November 2018) in Coventry, UK (52.4128° N, 1.5090° W), particularly in overweight and obese participants. Ninety percentage of the participants could not achieve dietary VitD intake of the recommended 400 IU/day. VitD supplementation at 1,000 IU/day for 12 weeks significantly increased plasma 25(OH)D levels but was not sufficient to increase 25(OH)D to the desired level of 75 nmol/L (25) in this population (none achieved this) and had no significant effects on the metabolic and immunological parameters measured in the study apart from the reduction of plasma creatinine concentration in VitD group compared with the control group. Dietary VitD intake was kept unchanged during the study period in both groups.

The average plasma 25(OH)D was 37.0 ± 6.4 nmol/L for all participants at baseline with 33% participants categorized as VitD deficient, 57% as insufficient, and 10% sufficient. This was echoed by National Diet and Nutrition Survey (NDNS) data combined between 2008 and 2012 that the plasma 25(OH)D was 43.5 ± 1.68 nmol/L in 65 plus years old, among which 23% were VitD deficiency, 65% insufficiency and 10% sufficiency (26). Even though applying a low threshold of plasma 25(OH)D at 25 nmol/L, SACN (22) clearly states that 30–40% of the UK population are below this concentration in winter versus 2–13% in summer due to different sunlight exposure. Even though VitD deficiency is prevalent in elderly population, a greater significance is observed in children (4–10 years) and teenagers (11–18 years) (26). However, this can be explained by higher use of VitD supplementation in the elderly (24–32%), compared to teenagers (5–6%) and children and adults (14–19%) (26). In the current study, none of the participants took 400 IU/day or over of VitD supplementation.

At baseline, there was a significant inverse association of plasma 25(OH)D concentrations and BMIs, and a significant lower mean plasma 25(OH)D (22.3 nmol/L) in overweight/obese participants than that in the normal weight (48.1 nmol/L), which supports the observation that obesity has been associated with lower 25(OH)D concentrations (27). Volumetric dilution is the most accepted explanation (28), while VitD, being fat soluble, can also be stored in cutaneous and visceral adipose tissues, resulting in lower plasma VitD concentrations in overweight and obese individuals (28). It is still unclear whether VitD deficiency is a cause or an outcome of obesity, and it may be a complex of mutual influence because VitD receptors are expressed on adipose cells and have a role in the function of those cells (29).

VitD3 supplementation at 1,000 IU/day for 12 weeks significantly increased the mean plasma 25(OH)D concentration from 38.4 ± 5.4 nmol/L at baseline (V1) to 51.8 ± 5.8 nmol/L after 12 weeks (V3), though far less than the optimal of 75 nmol/L. Except for one participant in the VitD group who had a baseline 25(OH)D at 149 nmol/L, none of others achieved the optimal level at the end of the intervention. The increase in plasma concentration of 25(OH)D in the VitD group reached a plateau after 6 weeks of supplementation and was then stable until the end of the study. This result was supported by other studies. One shows supplementation with 800 IU/day VitD3 raised the mean serum 25(OH)D concentration from a mean baseline of 46.9 ± 20.6–71.4 ± 21.5 nmol/L at 3 months (no measure of 25(OH)D before 3 months) and was kept stable up to 2 years of supplementation (18). Another randomized, double-blinded controlled trial with oral administration of 400, 800, 1,200 or 2,000 IU/day of VitD3 for 16 weeks showed that the dose response of serum 25(OH)D concentration was curvilinear with a plateau around 6 weeks for all doses with higher dose reached higher serum 25(OH)D concentrations (30). A group of healthy adults were provided 2,000 IU/day VitD2 (*n* = 28) or VitD3 (*n* = 31) over 140 days and found that the plateau of plasma 25(OH)D was achieved at 56 days of the supplementation and kept stable till 140 days (31). Menopausal women who were provided VitD3 400, 800, 1,600, 2,400, 3,200, 4,000, and 4,800 IU/day for a year and the plasma 25(OH)D did not distinguish after 6 months and 12 months of supplementation for all doses with higher dose reaching higher serum 25(OH)D concentrations (32). This observation of plateau of the serum 25(OH)D after VitD supplementation can be due to the fact that at a lower concentration of 25(OH)D, the conversion of VitD3 from oral or cutaneous intake into 25(OH)D follows first-order kinetics, which later switches to a slower, zero-order with saturation of hepatic 25-hydroxylase, and the 25(OH)D levels start to plateau (33). This observation indicates that in order to achieve the optimal 25(OH)D level, a higher dose is needed for longer period of supplementation rather than lower dose but prolonged period of supplementation.

Human observational studies have consistently reported inverse association of VitD status with inflammatory markers including C-reactive protein (CRP), IL-6 and TNF-α in healthy older populations (6, 34), and in those with proinflammatory conditions, such as diabetes, arteriosclerosis, asthma, inflammatory bowel disease, and chronic kidney disease (35). However, the current study does not provide complementary results to the above studies, which is likely due to the low statistical power and low dose of supplementation. However, this result was supported by other studies. A randomized controlled trial (RCT) of 3-weeks’ VitD supplementation of 2,000 IU/day did not change the resting plasma TNF-α concentration in a cohort of runners (36), and another RCT administering 100,000 IU/15 days of VitD3 for 3 months in older population did not have any significant impact on plasma levels of TNF-α, IL-6 and transforming growth factor β (TGF-β) (37). Interestingly, Żebrowska (36) found that VitD supplementation significantly reduced 1 h post-exercise TNF-α levels when the TNF-α concentration was increased compared with that in the rest, and Goncalves-Mendes et al. (37) observed significant lower plasma TNF-α and IL-6 level, higher TGF-β level 28 days post influenza vaccination administrated after the 3 months of VitD3 supplementation (100,000 IU/15 days) in older adults. A systematic review and meta-analysis by Wang et al. (38) including 20 RCTs of 1,464 patients with diabetic nephropathy, found that VitD supplementation significantly decreased CRP, IL-6 and TNF-α. This implies that an anti-inflammatory effects of VitD supplementation may exert their effects in situations of physiological stress or challenging situations when the proinflammatory cytokines are increased rather than simply lowering basal levels at rest.

There are very limited studies investigating the effect of VitD on phagocytosis of neutrophils and monocytes and lymphocytes subsets. An *in vitro* experiment using macrophages derived from monocytes of the healthy adults shows that active form of VitD3 (1,25 (OH)D3) at 100 nM (10^–7^M) significantly upregulated the macrophage complement receptor immunoglobulin which has been found to play a key role in the phagocytosis and clearance of bacteria (39). Another *in vitro* study found that the active form of VitD3 at a concentration of 10^–7^ M significantly enhanced the macrophage phagocytosis of live *M. tuberculosis* in normal adults with low phagocytic potential (less than 10%), but not in pulmonary tuberculosis patients (40). However, evidence from RCTs did not support the *in vitro* results. The current study found no significant difference in phagocytic activity of granulocytes and monocytes, which was supported by Goncalves-Mendes et al. (37) that receiving a much higher dose of 100,000 IU/15 days of VitD3 supplementation over a 3-month period significantly increased serum 25(OH)D from 51.8 ± 14.3 to 110.8 ± 21.5 nmol/L but did not change the phagocyte reactive oxygen species (ROS) production compared with the placebo group in the older adults with plasma 25(OH)D < 75 nmol/L at baseline. Regarding lymphocyte subsets, the current study did not observe any changes in proportion of Th, Tc, and NK cells, while a supplementation of VitD3 at a much higher dose (100,000 IU/15 days) (37) did not change the proportion of T-cells, Th, activated T-cells, Th1, Th2, Th17, and T regulatory (Treg) cells in older adults. Another RCT conducted in 60 healthy adults (24–42 years old), who consumed high-dose VitD3 supplementation (140,000 IU/month) for 12 weeks showed the 25(OH)D concentration was increased from an average 63.8 nmol/L at baseline to 137.8 nmol/L, but had no effect on the frequency of monocytes, dendritic cells, NK cells, NK T cells, B cells and subgroups of T cells (41). Though it seems that VitD supplementation may not change lymphocyte subset frequency, some evidence show that VitD supplementation had an impact on Treg cells. For example, the earlier mentioned RCT by Prietl et al. (41) showed a significant increase in the number of the peripheral Treg cells after VitD supplementation in healthy adults. A few trials in patients with auto-immune diseases showed an increase in circulating Treg cell proportion after 12 months of VitD supplementation in asthma children at a dose of 650 IU/day (42), and 1,000 IU/week (43), and in Type 1 diabetes at 2,000 IU/day (44) according to a systematic review (45). The clinical implication of the increase in the proportion of the Treg cells warrants further investigation.

It is of interest to explore how VitD supplementation would affect the severity of symptoms of participants who experience a significant immunological challenge such as respiratory infection, given what were stated earlier that the effect of VitD supplementation on the immune system might be more manifested when immune system is challenged. A RCT of 5,000 IU/day of vitamin D3 supplementation during 4 weeks of winter training to young taekwondo athletes (*n* = 13) with vitamin D insufficiency (25(OH)D < 50 nmol/L) significantly increased serum 25(OH)D concentration? from 28.7 ± 1.51 to 100.1 ± 4.70 nmol/L and caused a significant reduction in upper respiratory tract infection (URTI) symptom scores, but no significant increase in salivary sIgA concentration compared to placebo (*n* = 12) (46). Though this was a small study, it was aligned with a systematic review by Martineau et al. (47) that evaluated 10,933 participants in 25 RCTs and found a significant overall reduction in acute respiratory infections following vitamin D supplementation (OR 0.88, 95% CI 0.81–0.96) compared with no supplementation. A recent review of evidence from human studies on the health effects of VitD supplementation (48) concluded that supplementation of VitD-replete individuals does not generate overall health benefits including the incidence of cancer, cardiovascular events or type 2 diabetes mellitus (T2DM), bone density and the risk of falls, it did find that some beneficial effect on the respiratory tract infection and COVID-19 severity. However, the immunological mechanisms of the effect are unknown, which is worthwhile further research.

The current study found a significant reduction in plasma creatinine in VitD supplement group compared with the control group. The serum creatinine level tends to represent the renal functions. All participants were within the normal plasma creatinine concentrations. Though limited data are available in this area, a cross-sectional study found a significant negative correlation between serum 25(OH)D and creatinine concentrations in 60 Saudi Arabian patients with end stage renal disease (49). A recently published large-scaled randomized, double-blind, placebo-controlled trial (*n* = 545) found that a daily oral supplementation of 2,000 IU of VitD3 plus 600 mg calcium for 6 months in free living adults in United Arab Emirates significantly increased the ratio of calcium and creatinine compared with VitD3 or calcium supplementation alone (50). The mechanism of the possible protective role that VitD may play in kidney disease is thought due to VitD’s suppression of the renin-angiotensin-aldosterone system (RAAS) leading to improved estimated glomerular filtration rate (eGFR) (51). However, some evidence showed different outcomes. In an American study, 16 patients with chronic kidney diseases were provided VitD receptor activator, paricalcitol 2 μg a day for 7 consecutive days and the results showed a significant increase in serum creatinine concentrations without changing glomerular filtration rate (52). The mechanisms speculated by the authors might be due to the increased creatinine generation by muscle mass because VitD receptors exist in muscle cells (52). The aforementioned systematic review and meta-analysis by Wang et al. (38) found that VitD supplementation provides beneficial effects on 24-h urine protein and inflammation indexes, but not on serum creatinine concentration and eGFR. A mendelian randomization study based on three single nucleotide variants associated with 25(OH)D levels: rs2282679, rs10741657, and rs12785878, related to the genes GC, CYP2R1 and DHCR7, respectively, found that a 10% increase in serum 25(OH)D levels causes a 0.3% decrease in eGFR (53), while there was no effect of VitD supplementation of 4,000 IU per day versus placebo for a median duration of 2.9 years on changes in eGFR in adults with prediabetes (54), though there were no data of serum creatinine available in the above two studies. The above conflicting results are worthy of further research regarding VitD supplementation, blood creatinine, and kidney function.

Our results did not observe an increase in either plasma 25(OH)D concentration or dietary VitD intake in the control group over 12 weeks of study period with initial education. This indicates an unsuccessful attempt of educating participants to improve their VitD status via dietary VitD intake and sun exposure in the absence of VitD supplementation, which supports the advocate of applying food fortification to combat VitD deficiency (55) due to adherence issue as well as potential overdosing of VitD supplements (56, 57).

### Limitation of study

There are a few limitations of the study. Firstly, this was a pilot study with a small sample size thus low statistical power. Low statistical power increases the chance of type II error, or false negative and increases the likelihood of inflated effect (58). For example, the ratio of CD4/CD8 tended to be lower in VitD group compared with the control group, though there was no significant difference (*P* = 0.168), similar results were observed for IL-6 (*P* = 0.296) and CD56 (*P* = 0.212) (Table 6), possibly due to the low statistical power. Future research is required with an appropriate sample size with power calculation, higher VitD dose and a longer period of intervention (at least 6 months) to assess the health benefit of VitD on immune function. Secondly, evidence indicates that VitD supplementation is most likely efficient only in deficient individuals (59). The current study did not exclusively recruit VitD deficient participants, however, all of the participants in the study were VitD insufficient (25(OH)D < 50 nmol/L apart from one (25(OH)D = 149 nmol/L at baseline). Thirdly, participants were only asked to consume one table per day with meals, and no specific guidelines of VitD3 tablet intake was provided. VitD is a fat-soluble vitamin, therefore, it is recommended to be consumed ideally with a fat-rich meal because dietary fat increases VitD3 absorption (60). In addition, evidence shows there was an inverse relationship of 25(OH)D concentrations and melatonin (a hormone important in regulating the circadian cycle) (61), therefore, VitD3 tablets are preferably taken in the morning with meals so that levels of blood 25(OH)D are higher during the day, mimicking what is observed naturally and avoid taking the tablets in the evening or at night. In addition, further research in VitD supplementation and immune function should include parathyroid hormone (PTH) and the glycated hemoglobin A1c (HbA1c) tests. PTH and VitD form a tightly controlled feedback cycle to maintain calcium and phosphate homeostasis, with PTH being a major stimulator of vitamin D synthesis in the kidney while vitamin D exerts negative feedback on PTH secretion (62). PTH is also an immunologic mediator because PTH receptors were found on most immunologic cells including neutrophils, B and T cells (63) and can also be used as an indicator of Vit D resistance assuming adequate dietary calcium and phosphate intake and ruling out of a differential diagnosis of hyperparathyroidism (64). HbA1c is an overall marker of the average blood glucose levels over a period of 2–3 months (65). A systematic review and meta-analysis (66) including 19 RCT studies involving 747 intervention subjects and 627 placebo controls revealed that VitD supplementation significantly reduced HbA1c but not fasting blood glucose in people with type 2 diabetes, compared with placebo.

In conclusion, VitD deficiency is common among older population (aged 55–85 years) even during the summer/autumn time in the UK. 90% of the participants did not meet dietary VitD intake recommendation of 10 μg/day. Overweight/obese participants had a significant lower plasma 25(OH)D than normal weight counterparts. VitD supplementation at 1,000 IU/day for 12 weeks significantly increased plasma 25(OH)D levels in the first 6 weeks which was kept stable till 12 weeks. However, the dose could not increase 25(OH)D to the desired level of 75 nmol/L. The dose of VitD supplementation might be too low to have impact on metabolic and immunological parameters measured in the study apart from creatinine. There was no difference in dietary VitD intake and plasma 25(OH)D concentrations between baseline and 12 weeks of intervention in the control group, indicating an unsuccessful attempt of education in the current study.

## Data availability statement

The raw data supporting the conclusions of this article will be made available by the authors, without undue reservation.

## Ethics statement

The studies involving human participants were reviewed and approved by Coventry University Ethics Committee (Reference no. P66509), Coventry University, United Kingdom. The patients/participants provided their written informed consent to participate in this study.

## Author contributions

HD, SF, and DR designed the study. HD conducted the trial. SF and DR provided guidance and support to the trial. HD and VA tested the blood samples. HD analyzed data and produced the manuscript. All authors reviewed and agreed with the manuscript.
