# Supplementary material for: Influence of vitamin D supplementation on immune function of healthy aging people: A pilot randomized controlled trial
Source: Front Nutr. 2022 Nov 1;9:1005786. doi: 10.3389/fnut.2022.1005786 (PMC9664161; doi:10.3389/fnut.2022.1005786)
Supplement: Supplementary file 1 [file Data_Sheet_1.docx]

Supplementary Material

Table S1 Health and Lifestyle Questionnaire

| **Name (initials):** | **Height (m):** |
| --- | --- |
| **Age:** | **Weight (kg):** |
| **Gender:** | **Ethnicity:** |
| **Contact number:** | **Email:** |

| Have you been suffering from digestive disease that may affect nutrient intake? | YES / NO | *If yes, please give details.* |
| --- | --- | --- |
| Have you been diagnosed as having diabetes? | YES / NO |  |
| Do you have liver or kidney disease? | YES / NO |  |
| Do you suffer from any other illness? | YES / NO | *If yes, please give details.* |
| Are you currently on any long-term medication? | YES / NO | *If yes, please give details.* |
| Do you smoke? | YES / NO |  |
| Do you drink alcohol? | YES / NO | *If yes approximately how many units per week do you drink?* |
| Are you gluten intolerant? | YES / NO |  |
| Are you vegetarian? | YES / NO |  |
| Do you take any form of dietary supplement e.g. fish oils, vitamins or minerals? | YES / NO | *If yes, please give details.* |

Table S2 The reported and actual intake of vitamin Ds supplement tablets

| Participant | Reported intake (n) | Actual intake (n) |
| --- | --- | --- |
| 1 | 83 | 84 |
| 2 | 72 | 70 |
| 3 | 83 | 80 |
| 4 | 84 | 78 |
| 5 | 84 | 85 |
| 6 | 84 | 86 |
| 7 | 84 | 83 |
| 8 | 84 | 85 |
| 9 | 83 | 80 |
| 10 | 84 | 74 |
| 11 | 84 | 85 |
| 12 | 84 | 84 |
| Mean ± SE | 82.8 ± 1.0 | 81.2 ± 1.4 |
